# Extraction Optimization, Structural Analysis, and Potential Bioactivities of a Novel Polysaccharide from *Sporisorium reilianum*

**DOI:** 10.3390/antiox13080965

**Published:** 2024-08-08

**Authors:** He Shi, Siyi Zhang, Mandi Zhu, Xiaoyan Li, Weiguang Jie, Lianbao Kan

**Affiliations:** 1 Engineering Research Center of Agricultural Microbiology Technology, Ministry of Education, Heilongjiang Provincial Key Laboratory of Plant Genetic Engineering and Biological Fermentation Engineering for Cold Region, Key Laboratory of Microbiology, College of Heilongjiang Province, School of Life Sciences, Heilongjiang University, Harbin 150080, China; shihe0112@163.com (H.S.); zhangsiyi11u@163.com (S.Z.); 13343088849@163.com (M.Z.); 2 School of Life Sciences, Northeast Forestry University, Harbin 150040, China; xyli821187@163.com

**Keywords:** *Sporisorium reilianum*, polysaccharide, antioxidant activity, antitumor activity

## Abstract

*Sporisorium reilianum* is an important biotrophic pathogen that causes head smut disease. Polysaccharides extracted from diseased sorghum heads by *Sporisorium reilianum* exhibit significant medicinal and edible value. However, the structure and biological activities of these novel polysaccharides have not been explored. In this study, a novel polysaccharide (WM-NP’-60) was isolated and purified from the fruit bodies of *S. reilianum* and aimed to explore the structural characteristics and substantial antioxidant and antitumor properties of WM-NP’-60. Monosaccharide composition determination, periodate oxidation-Smith degradation, 1D/2D-NMR analysis, and methylation analysis revealed that WM-NP’-60 consisted mainly of *β*-1,6-D-Glc*p*, *β*-1,3-D-Glc*p*, and *β*-1,3,6-D-Glc*p* linkages. The antioxidant assays demonstrated that WM-NP’-60 exhibited great activities, including scavenging free radicals, chelating ferrous ions, and eliminating reactive oxygen species (ROS) within cells. The HepG2, SGC7901, and HCT116 cells examined by transmission electron microscopy (TEM) revealed typical apoptotic bodies. Therefore, a novel fungal polysaccharide (WM-NP’-60) was discovered, extracted, and purified in this experiment, with the aim of providing a reference for the development of a new generation of food and nutraceutical products suitable for human consumption.

## 1. Introduction

Polysaccharides possess the ability to protect neuronal cells from cellular toxicity induced by free radicals, making them widely used in the preparation of therapeutic agents for neuronal diseases [1,2]. Cancer, atherosclerosis, inflammation, and senescence are only some of the human disorders linked to oxidative stress, which can be induced either directly or indirectly by reactive oxygen species (ROS). Antioxidants can disrupt the oxidative reactions caused by free radicals by interrupting chain reactions and providing hydrogen atoms, thereby preventing diseases associated with oxidative damage [3]. However, synthetic antioxidants may have side effects such as liver damage and carcinogenicity [4]. Therefore, the search for novel antioxidants from natural sources is crucial in protecting the human body from free radical damage without producing adverse effects.

Fungal polysaccharides possess various biological activities, including immune stimulation, antitumor, antiviral, antioxidant, and hypoglycemic effects [5,6,7,8,9]. The potential of mushroom polysaccharides in the food and pharmaceutical industries is rising. Several fungal polysaccharides have demonstrated potent antioxidant and antitumor activities, which are closely associated with their health-protective functions [10,11,12]. Previous studies have found that WM-NP-60 exhibits excellent proliferation of HepG2 and SGC7901 cells can be inhibited [13]. Therefore, fungal polysaccharides have garnered significant interest in the food and pharmaceutical industries for the development of non-toxic natural antioxidants and antitumor drugs, which offer notable health benefits.

The sorghum head sumt is the result of infection by *Sporisorium reilianum*, also known as “sorghum Wu mi” in China. The sorghum head blight is a major problem all across the world [14]. From the perspective of traditional Chinese medicine, however, the “Wu mi” also has several nutritious characteristics. Sorghum is a valuable crop for addressing global climate change and population growth. Many nutrients, including carbohydrates, vital amino acids, vitamins, and so on, can be found in *S. reilianum* [15]. Only the Chinese Pharmacopoeia includes *S. reilianum* in its database. In fact, *S. reilianum* has been used as folk medicine and food for many years [16,17]. In this study, the polysaccharides WM-NP’-60 were purified from *S. reilianum* fruit bodies. Preliminary structural features of the WM-NP’-60 have been determined and antioxidant activities and antitumor activities were evaluated, which provided a theoretical foundation for applying WM-NP’-60 in the food and pharmaceutical field.

## 2. Materials and Methods

### 2.1. Materials

Heilongjiang University’s College of Life Sciences is where voucher specimens are kept. 1,2,3-phenyltriol, potassium ferricyanide [K_3_Fe(CN)_6_], horseradish peroxidase (HRPase), 2-thiobarbituric acid (TBA), 1,1-diphenyl-2-picrobutylhydrazine (DPPH), trichloroacetic acid (TCA), nitroblue tetrazolium salt (NBT), ascorbic acid, ferrizine, phenazine mesylate (PMS), dihydronicotinamide adenine dinucleotide (NADH), thiobarbituric acid (TBA) and deoxyribose, and ethylenediamine tetraacetic acid (EDTA) were purchased from Sigma Corporation (St. Louis, MO, USA).

### 2.2. Polysaccharide Extraction

The dried sorghum Wumi was processed into fine powder (particle size 40 mesh) and extracted with hot water to obtain the polysaccharides. Finally, the polysaccharides were separated from the extract by ethanol precipitation; 20 L of 95% ethanol was used to extract 2 kg of sorghum Wumi. After mixing the sorghum Wumi with ethanol, the sorghum Wumi was placed for 24 h in the extraction process to achieve a full extraction effect. The extracted mixture was then degreased to remove the fat components. The dried residue will be washed three times with 20 times the volume of distilled water at 80 °C and then filtered. The filtrate was merged and concentrated by polyethylene glycol dialysis, after which the Sevag method was used for protein removal [18]. The water extract needed to go through a centrifugation process at the rotating speed of 8000 rpm; the centrifugation process lasted for 5 min. After being kept at 4 °C for 24 h, the supernatant was combined with ethanol to reach a final concentration of 80%.

**Figure 1:**
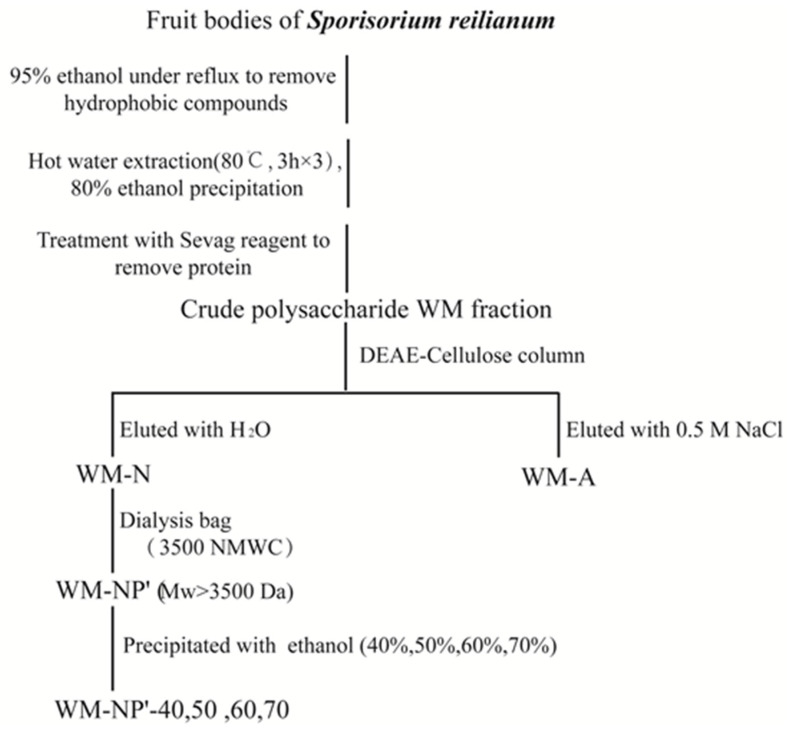
Procedure for the extraction and purification of *S. reilianum* polysaccharides.

Following three washes with anhydrous ethanol, acetone, and ether, it was centrifuged at a speed of 3000 rpm for 15 min to obtain a precipitate. In total, 184 g of crude polysaccharide (WM) was successfully obtained by the freeze-drying process. Figure 1 shows the method of extracting and fractionating polysaccharides from *S. reilianum*.

### 2.3. Extraction Optimization on S. reilianum Polysaccharides

#### 2.3.1. Single Factor Experiments

Experiments with a single variable were carried out to determine the impact of factors such as extraction temperature, extraction time, solid–liquid ratio, and extraction frequency. In the experiment, selected different extraction temperatures (60 °C, 70 °C, 80 °C, 90 °C, and 100 °C), extraction time (60, 120, 180, 240, and 300 min), solid–liquid ratio (1:5, 1:10, 1:20, 1:40, and 1:60 *w*/*v*), and extraction frequency (1, 2, 3, 4, and 5) were the independent variables. Each experiment was conducted three times with the WM yield serving as the detection index.

#### 2.3.2. Orthogonal Test Design

On the basis of the single-factor experiment, the L_9_ (3^4^) orthogonal test was used to test the four factors of Table 1. In order to obtain accurate results, a total of 9 experiments were carried out shown in Table 2.

### 2.4. Purification of S. reilianum Polysaccharides

#### 2.4.1. Analytical Chromatography on DEAE-Cellulose

The crude WM was completely dissolved and injected into a DEAE-cellulose column (9.8 × 30 cm). Then, the elution was carried out sequentially. During the elution process, the flow velocity was 20 (mL/min). Two main final components (WM-N and WM-A) were obtained. The mixture was separated, salted, dialyzed, freeze-dried, and stored at 20 °C. Through these steps, 184 g of WM polysaccharides were successfully purified.

#### 2.4.2. Dialysis Fractionation of WM-N Polysaccharide

The WM-N with a mass of 80 g was completely dissolved in a certain amount of distilled water, it was ensured that this dissolved thoroughly via magnetic stirring. The liquid was transferred to a dialysis bag with 3500 Da interception molecular weight (*Mw*) and put into distilled water for dialysis. The distilled water should be replaced every 3 h, before replacement, 1 mL of dialysis fluid should be removed from the outer pouch, and phenol sulphuric acid should be used to measure the sugar. The dialysis process should be stopped until the reaction color of the sugar cannot be observed. Then, the WM-NP’ component in the collection bag (*Mw* > 3500 Da) was concentrated and freeze-dried. After that, the sample was weighed and its sugar content, protein content, monosaccharide composition, and relative *Mw* were determined.

#### 2.4.3. Alcohol Precipitation Fractionation of WM-NP’ Polysaccharides

WM-NP’ polysaccharides were mixed aqueous solution (10 mg/mL) and completely dissolved after 24 h of stirring. Next, in the process of slowly adding anhydrous ethanol, the concentration of ethanol in the solution reached 40%. After waiting for 24 h, the WM-NP’-40 was centrifuged and precipitated to keep it dry. Then, under the same conditions, only the final concentration of ethanol was changed and the polysaccharides precipitation of ethanol concentrations of 50%, 60%, and 70% were obtained. The above precipitates were weighed and their relative *Mw* and sugar content were determined.

WM-NP’ solutions (10 mg/mL, 20 mg/mL, 50 mg/mL) were prepared. Then, by adding an appropriate amount of absolute concentration of ethanol to the WM-NP’ solution, the final concentration of ethanol reached 60%. The ethanol precipitates obtained under different sample concentrations were accurately weighed and the sugar content was determined.

### 2.5. Determination of the Relevant Properties of S. reilianum Polysaccharides

#### 2.5.1. Determination of Sugar Content

The experiment was carried out to measure the sugar content of polysaccharides by the phenol-sulfuric acid method [19]. The standard, which used a solution of glucose (0.1 g/L), was prepared and different volumes of the standard (0.1 g/L) were placed in 5.0 mL colorimeter tubes. The solutions were diluted to the scale line with distilled water. Then, it was added in turn with 6% phenol (0.5 mL) and concentrated with H_2_SO_4_ (2.5 mL), mixed well, and left to stand for 10 min. The absorbance of each solution was assessed at *OD*_490nm_ after zeroing with a blank test. A standard curve was constructed with the mass of glucose on the horizontal axis and its corresponding absorbance value on the vertical axis. The experiment was repeated three times. For the determination of the samples, to the measured polysaccharide sample solution (1 mL), 6% phenol (0.5 mL) and concentrated H_2_SO_4_ (2.5 mL) were added in turn. After thorough mixing and allowing it to stand for 10 min, the absorbance value was measured. Subsequently, the sugar content of the sample was determined.

#### 2.5.2. Determination of Protein Content

The experiment was carried out to measure the protein content of polysaccharides by the Kaomas Brilliant Blue method [20]. Different volumes of the standard protein solution (50 μg/mL) were put into 5.0 mL colorimetric tubes and diluted to the scale with distilled water. Then, Kaomas Brilliant Blue (1 mL) was added and left to stand for 5 min. The absorbance value of each solution was determined at *OD*_595nm_. A blank test was zeroed. The standard curve was plotted with the content of bovine serum albumin as the horizontal coordinate and the corresponding absorbance value as the vertical coordinate. The experiment was repeated three times. For the determination of the samples, the measured polysaccharide sample solution (200 μL) was accurately measured with a pipette, Koammas Brilliant Blue (1 mL) was added, mixed well, and left to stand for 5 min. Subsequently, the absorbance values were determined following the standard curve plotting protocol. The protein content of the sample was then quantified.

#### 2.5.3. Determination of Glucuronic Acid Content

The m-hydroxybiphenyl method was used to measure the glucuronic acid content of polysaccharides [21]. Different volumes of D-GalA standard solution (0.1 g/L) were placed in 5.0 mL colorimetric tubes and then diluted to the scale with distilled water. Aminosulphonic acid (40 μL) was added in turn and shaken gently. Then, it was mixed well with concentrated H_2_SO_4_ (2.5 mL), heated, and boiled for 20 min. It was then mixed well with m-hydroxybiphenyl (40 μL) at room temperature and then left for 15 min. The solutions were analyzed for absorbance values by ultraviolet spectrophotometer at *OD*_525nm_. The standard curve was plotted with the content of D-GalA as the horizontal coordinate and the corresponding absorbance value as the vertical coordinate. The experiment was repeated three times. For the determination of the samples, the polysaccharide sample solution (0.4 mL) was measured accurately and aminosulfonic acid (40 μL) was added sequentially with gentle shaking. It was mixed well with concentrated H_2_SO_4_ (2.5 mL). The m-hydroxybiphenyl (40 μL) was added at room temperature and mixed well after being heated and boiled for 20 min and then cooled down. The absorbance value was measured by following the procedure of the standard curve plotting. The glucuronide content of the samples was then calculated.

#### 2.5.4. Monosaccharide Composition Determination of Polysaccharides

The monosaccharide composition was determined by HPLC after precolumn derivatization of the hydrolysate with PMP [22]. In short, the polysaccharide was hydrolyzed using 2.0 mol/L trifluoroacetic acid (TFA) at 120 °C for 1 h in a sealed tube. Any remaining excess acid was eliminated by drying at 45 °C with the addition of a small amount of ethanol post-hydrolysis. Following this, the dry hydrolysate samples or a standard aqueous solution of monosaccharides were combined with a 0.5 mol/L methanol solution of PMP (500 µL) and 0.3 M aqueous NaOH (500 µL) for derivatization at 70 °C for 30 min. Subsequently, the mixture was centrifuged at 10,000 rpm for 5 min. HCl (50 µL, 0.3 mol/L) was introduced to the mixture solution, followed by extraction with chloroform. The resulting aqueous layer was then filtered through a 0.22 µm membrane for subsequent HPLC analysis. Subsequently, the filtrate (10 µL) was injected into a DIKMA Inertsil ODS-3 column (4.6 × 150 mm). PMP-tagged monosaccharide analysis was conducted using a Shimadzu LC-10ATvp HPLC system from SHIMADZU in Kyoto, Japan, which was outfitted with a UV detector set at *OD*_245nm_. The mobile phase consisted of a blend of PBS (0.1 mol/L, pH 7.0) and acetonitrile (81:19) and elution was performed at a flow rate of 1.0 mL/min.

#### 2.5.5. Molecular Weight Determination of Polysaccharides

The *Mw* of the polysaccharides was assessed using high-performance gel permeation chromatography (HPGPC) employing a TSK-gel G-3000PWXL column (7.8 × 300 mm) and a refractive index detector (Shimadzu RID-10A). The column temperature was held constant at 40 °C and the samples were eluted with 0.2 mol/L NaCl at a flow rate of 0.6 mL/min. The molecular weight was approximated by comparing it to a calibration curve generated using dextran standards. A set of dextran standards of various molecular weights (50,000, 25,000, 12,000, 5000, and 1300 Da) were used for the calibration curve. The retention times were 11.492, 12.105, 12.752, 13.525, and 14.715 min, respectively. The information was collected and analyzed using the Shimadzu LC-20A Solution software.

### 2.6. Structural Analysis of WM-NP’-60

#### 2.6.1. The Oxidation of Periodates and the Degradation of Smith Analysis

In order to determine the chemical structure of polysaccharides, the periodate oxidation analysis method was used [23]. Firstly, 50 mg of the sample was dissolved in 20 mL of NaIO_4_ solution with a concentration of 0.015 mol/L and the solution was kept at 4 °C under dark conditions. The spectrophotometer was used to measure the absorption of the reaction solution at *OD*_223nm_ every 6 h in a serious manner. After the completion of the oxidation reaction (48 h), 0.1 mL of ethylene glycol was used to decompose the excess NaIO_4_. The amount of NaIO_4_ used was determined by measuring the absorbance drop at *OD*_223nm_. The determination of the amount of formic acid produced was carried out through the process of titration using a solution of 0.1 mol/L NaOH. Next, the reaction mixture was subjected to water and distilled water dialysis treatment for 48 h. It was then concentrated at 50 °C using a rotary evaporator and then reduced by KBH_4_ for 12 h. Neutralization of the solution to pH 7.0 was conducted, which was then subjected to undergo the above dialysis and concentration steps until the volume was reduced to 10 mL. The solution of the above-mentioned 1/3 was freeze-dried and then hydrolyzed at 120 °C for 2 h with 2 mol/L trifluoroacetic acid (CF_3_COOH) (1 mL). After being reduced by NaBH_4_, it was acetylated at 90 °C for 1 h with the acetylation reagents pyridine (0.5 mL) and acetic anhydride (0.5 mL). Finally, the composition of sugar was analyzed by gas chromatography (GC). The chromatographic column was Shimadzu GC-14C and the chromatographic column was RTX-2330 column (0.32 mm × 15 m i.d., 0.2 μm).

#### 2.6.2. 1D/2D-NMR Analysis

In order to carry out the experiment, a substance with a weight of 20 mg of the sample was dissolved in 99.8% D_2_O solution with a quantity of 0.5 mL. Subsequently, the sample was freeze-dried and dissolved in 0.5 mL of D_2_O again. Any excess sample was removed by centrifugation. The MestReNova 14 was used to record the data in the whole experiment.

#### 2.6.3. Methylation Analysis

The linkage bond type of WM-NP’-60 was identified through methylation analysis. WM-NP’-60 underwent methylation following the procedure outlined by Bagchi and Jayaram Kumar [24]. The dried polysaccharide (5 mg) was dissolved in dimethyl sulfoxide (DMSO, 0.5 mL) and stirred until a clear solution was obtained. The solution was supplemented with 240 mg of anhydrous NaOH and stirred for 30 min. Following this, methyl iodide (3.6 mL) was introduced to the solution and stirred for 7 min. Subsequently, the mixture was combined with an equal volume of chloroform and stirred for 30 min. After adding 6 mL of distilled water to halt the reaction, the mixture was washed thrice with distilled water. The methylation of the product was validated using FT-IR spectroscopy. Following this, the methylated samples underwent hydrolysis by exposure to an 85% formic acid solution (1 mL) at 100 °C for 4 h and TFA (2 mol/L, 2 mL) at 100 °C for 6 h. Subsequently, co-distillation with methanol was carried out. Anhydrous ethanol and distilled water were employed sequentially to eliminate any residual acid. The hydrolyzed product underwent reduction with NaBH_4_ for an overnight period, followed by acetylation using 500 µL of acetic anhydride and pyridine at 100 °C for 2 h. Subsequently, co-distillation with anhydrous ethanol was performed and the solution was evaporated to dryness to yield the final product. After dissolution in chloroform, the methylated derivatives were identified using GC-MS (GCMS-QP 2010, Shimadzu, Kyoto, Japan).

#### 2.6.4. Determination of Optical Rotation

The WM-NP’-60 sample (5 mg) was dissolved in 5 mL of distilled water and formulated to a concentration of 1 mg/mL. The specific rotation was measured in an automatic polarimeter using D sodium light at 28.7 ± 1 °C.

### 2.7. Antioxidant Activity Assay In Vitro

#### 2.7.1. DPPH Radical-Scavenging Activity Assay

The ability to scavenge DPPH radicals was measured using Shimada’s technique [25]. To simplify, DPPH (0.1 mM) solution in methanol was added to 2 mL of sample solution (0.125–4 mg/mL) and ascorbic acid was employed as a standard. After a thorough shaking, the ingredients were left in the dark for 15 min before being left at room temperature for another 20 min. Next, the concentration of light absorbed by the solution at *OD*_517nm_ was measured. The percentage of scavenging activity of DPPH free radical can be obtained according to the calculation formula: scavenging activity (%) = (1 − A_sample_/A_control_) × 100.

#### 2.7.2. Hydroxyl Radical-Scavenging Activity Assay

Following Halliwell’s [26] protocol, a 0.125–4 mg/mL sample solution in 0.1 mL of reaction buffer was combined with 0.125 mL of 10 mM H_2_O_2_, 0.125 mL of 2 mM ascorbic acid, and 0.1 mM ferric chloride. The mixture was incubated at 37 °C for 90 min. Then, 0.1 mL of 10 mM of hydrogen peroxide, 0.1 mL of 2 mM of vitamin C, and 0.1 mL of 1 mM of ferrous dichloride were added. By measuring the absorbance of the mixture at *OD*_532nm_, the hydroxyl radical scavenging activity (%) was calculated.

#### 2.7.3. Evaluation of the Capacity to Scavenge Superoxide Anions

In this study, the removal ability of superoxide anion radical by NADH-NBT-PMS system was measured [27]. In the experiment, 1 mL of a concentration of 300 μM NBT, 1 mL of NADH at a concentration of 936 μM, and 1 mL of PMS at a concentration of 120 μM were added in 100 mM phosphate buffer. After incubation at 25 °C for 5 min, the scavenging activity (%) of the mixed solution to superoxide anion radical was evaluated by measuring the absorbance at *OD*_560nm_.

#### 2.7.4. H_2_O_2_-Scavenging Activity Assay

Samples were tested for their ability to remove hydrogen peroxide (H_2_O_2_) using the Pick and Mizel techniques [28]. Following a 20 min incubation period at room temperature, sample solution (1 mL, 0.125–4 mg/mL) was mixed with 400 μL of 5 mM H_2_O_2_ solution. Distilled water was selected as the negative control, while ascorbic acid was employed as the positive control. At *OD*_610nm_, the mixture’s ability to scavenge hydrogen peroxide was measured.

#### 2.7.5. Analyzing the Chelating Action on Ferrous Ions

According to the report of Dinis, its chelating activity with ferrous ion was determined [29]. First, 1 mL of sample solution (0.125–15 mg/mL) was mixed with 0.1 mL 2 mM FeCl_2_ solution for 30 s and reacted with 0.2 mL 5 mM ferrous hydrazine at room temperature for 10 min. Distilled water and ascorbic acid were used as a reference. By measuring at *OD*_562nm_, the complex activity percentage was calculated.

### 2.8. Measurement of ROS Production in Cell

Intracellular ROS levels were measured under the detection kit noS0033S (Beyotime, Jiangsu, China). Upon oxidation by ROS, the nonfluorescent DCFH-DA was converted to the highly fluorescent DCF [30]. The DCFH-DA probe was applied to detect the intracellular ROS levels. Cells were seeded in six-well plates for 24 h. After treatment with different concentrations of WM-NP’-60 for 2 h, an oxidative damage model was established by taking the cells to H_2_O_2_ (200 μmol/L) for 2 h. Subsequently, the cells were incubated with H2DCF-DA (10 µM) in darkness at 37 °C for 30 min. The DCF fluorescence intensity was showed by fluorescence microscopy.

### 2.9. Antitumor Activity Assays In Vitro

#### 2.9.1. Cells and Cell Culture

HepG2 and hepatic cells were provided by Northeast Forestry University. SGC7901, HCT116, GES-1, and CCD-18Co were provided by Harbin Medical University. NCM460 was purchased by bhcell biotechnological Co., LTD. (Gimpo-si, Republic of Korea). HepG2, SGC7901, HCT116, hepatic cells, GES-1, CCD-18Co, and NCM460 were cultured in DEME medium supplemented with fetal bovine serum (10%) and antibiotics (100 U/mL penicillin together with 100 µg/mL streptomycin) with a 5% CO_2_ atmosphere at 37 °C in humidified incubator.

#### 2.9.2. Anti-Proliferation Activity Assay

The 3-(4,5-dimethyl-2-thiazolyl)-2,5-diphenyl-2-H-tetrazolium bromide (MTT) assay was used to detect cell growth [31]. After seeding logarithmically growing HepG2 cells, SGC7901 cells, HCT116 cells, and three normal cells, including hepatic cells, GES-1, and CCD-18Co, they spent 24 h in a 37 °C incubator. After the cells were incubated, the supernatant was discarded and 100 μL of reagents containing 1, 2, 4, 6, or 8 mg/mL of WM-NP’-60 were added to each well. The board was then re-incubated in a 37 °C incubator for 24, 48, and 72 h. The control group was also established at the same time. MTT (10 μL, 5 mg/mL) were plated into each well within 4 h of the conclusion of the culture. Once the liquid had been absorbed during the 4 h incubation period at 37 °C, 100 uL of dimethyl sulfoxide was added to each well. After 10 min of gentle shaking, an *OD*_490_ reading was taken.

#### 2.9.3. TEM Assay

Both HepG2, SGC7901, and HCT116 cells were cultured in this experiment and then treated with either WM-NP’-60 (4 mg/mL) DMEM for 48 h. Subsequently, TEM was utilized to observe the morphological changes in the cells [32].

### 2.10. Statistical Analysis

The method of dividing data into three groups. Statistical software SPSS24.0 was used for analysis of variance and *t*-test. Data were expressed using the average ± standard deviation (SD). (* *p* < 0.05 and ** *p* < 0.01).

## 3. Results

### 3.1. Single Factor Experimental Analysis

#### 3.1.1. Effects of Extraction Temperature on WM Yield

**Figure 2:**
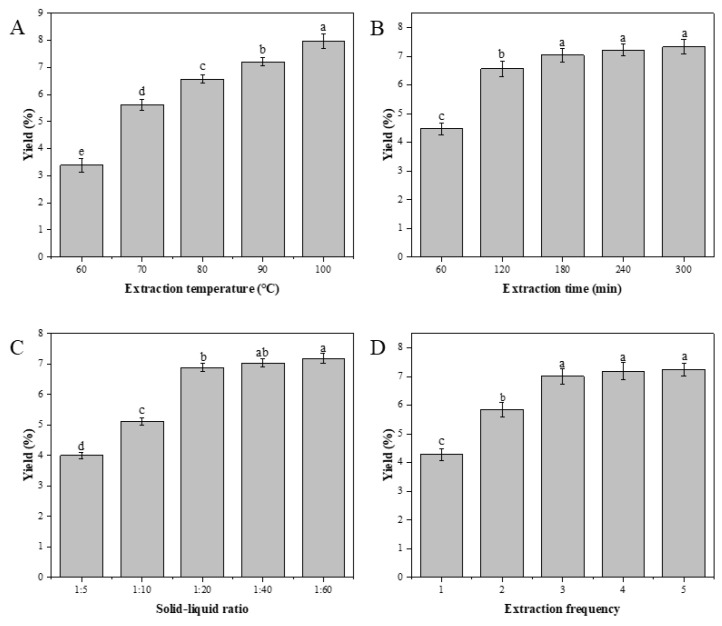
The yields of WM were evaluated under various extraction conditions, including temperature (**A**), time (**B**), solid–liquid ratio (**C**), and frequency (**D**). The effects of these factors on the yields were investigated. Note: different letters indicate significant differences (*p* < 0.05).

The effects of five different extraction temperatures (60, 70, 80, 90, and 100 °C) on the yield of WM polysaccharides were studied, under the proviso that all other experimental variables (such as extraction time, ratio of material to liquid, and extraction frequency) were held constant. Figure 2A shows that as temperatures rose, so did the output of WM polysaccharides. However, the increase in the yield of WM polysaccharides was not obvious when the extraction temperature increased from 90 °C to 100 °C. As an active substance, the polysaccharide’s structure and activity were easily impacted by high temperature and this resulted in an increase in price. Therefore, 80 °C was decided upon as the optimal temperature for the extraction process.

#### 3.1.2. Effects of Extraction Time on WM Yield

The influence of the extraction time (60, 120, 180, 240 and 300 min) on WM production was studied under the condition that other parameters (extracting temperature, solid liquid rate, and extracting frequency) were fixed. When the extraction time was less than 180 min, as illustrated in Figure 2B, the extraction time extension led to a notable increase in the yield of WM. When the duration of extraction surpassed 180 min, the extension of the extraction time only resulted in a minimal increase in the yield of WM. Therefore, the extraction time of approximately 180 min was deemed suitable.

#### 3.1.3. Effects of the Solid–Liquid Ratio on WM Yield

The yield of WM was studied in relation to the solid–liquid ratio, with all other experimental variables (extraction temperature, extraction time, and extraction frequency) held constant. The experiments were carried out at 1:5, 1:10, 1:20, 1:40 and 1:60, respectively. It was found that the production rate of polysaccharide was improved with the addition of aqueous solution. According to Figure 2C, there was a notable increase in polysaccharide yield as the solid-to-liquid ratio was elevated from 1:5 (g/mL) to 1:20 (g/mL). After 1:40 (g/mL), the increase rate slowed down significantly. Considering that the amount of liquid was too large, it will cause trouble for the subsequent process. The active ingredients of WM can easily be destroyed through long-term concentration, which consumes more energy. All things were considered; the solid–liquid ratio was preferably about 1:20 (g/mL).

#### 3.1.4. Effects of Extraction Frequency on WM Yield

Figure 2D showed a steady rise in yield as the extraction frequency was increased from 1 to 3. The production of polysaccharides was significantly improved during the extraction. When the rate of extraction was increased from three to five, the polysaccharide extraction yield increased very slowly. Because the extraction frequency exceeded 3, it was not significant to increase the extraction yield of polysaccharides. Therefore, three times was preferable.

### 3.2. Orthogonal Optimization Analysis

In a broad sense, the range analysis was called an intuitive analysis method. This method had many advantages, such as simple calculation, intuitive visualization, and ease of understanding. Therefore, the range analysis method was also selected in the experiment. A higher *R*-value indicates that a given component has a larger impact on the overall extraction efficiency. Experiment results showed that among the variables that affected extraction efficacy, temperature played the most important role, followed by the solid–liquid ratio, extraction frequency, and extraction time. The optimum process combination was identified as A_3_B_3_C_3_D_3_. Additionally, increasing the extraction temperature from 80 °C to 90 °C did not significantly enhance the extraction rate. Therefore, considering the structure, function, energy, efficiency, and cost of polysaccharides, it was concluded that the best extraction conditions for watermelon polysaccharides were A_2_B_2_C_2_D_2_. According to these parameters, the extraction conditions were as follows: 80 °C, 180 min, 1:20 solid–liquid ratio, and the extraction frequency was three.

**Figure 3:**
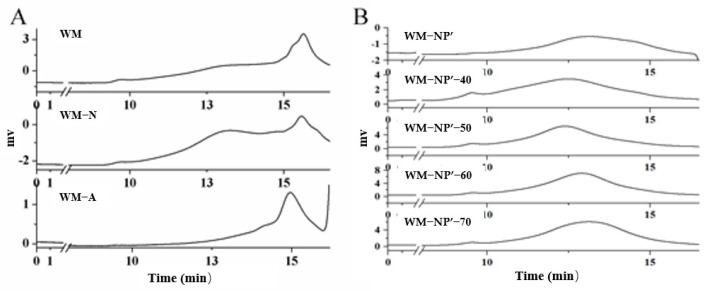
(**A**) HPGPC chromatogram of WM, WM-N, and WM-A; (**B**) HPGPC chromatogram of WM-NP’ products with different alcohol concentrations.

WM was the name given to the water-soluble polysaccharide isolated from the Wumi fruiting body. WM included 47.8% sugar, 6.1% glucuronic acid, and less than 1.2% protein. The results of the monosaccharide composition are shown in Table 3, the molar ratio of glucose to mannose to galactose to galacturonic acid to arabinose to glucuronic acid in WM polysaccharides is 69.6:15:11:1.9:1.7:0.8. (Related Figures S1–S3 and Tables S1–S3 are shown in the Supplementary Material). Zhao et al., used HPGPC to measure the *Mw* distribution of polysaccharides from *Polygonatum* spp. and found that the polysaccharides from *Polygonatum sibiricum* (PS), *P. cyrtonema* (PC), and *P. kingianum* (PK) showed two main peaks, whereas the polysaccharide from *P. odoratum* (PO) showed only one main peak. The results indicated that the *Mw* distribution of PS PC PK polysaccharides was not homogeneous [33]. In this study, the *Mw* of WM polysaccharide was detected as shown in Figure 3A; there was a notable variation in the *Mw* of WM polysaccharide, exhibiting a broad range and a higher abundance of oligosaccharides.

### 3.3. Fractionation by DEAE-Cellulose Chromatography

The 184 g WM polysaccharides were separated by chromatography with a DEAE- cellulose ion exchange column. After elution with distilled water, the weight of neutral sugar component WM-N was 94.3 g and the yield was 51.30%. Upon further elution with 0.5 M NaCl, the acid sugar component WM-A was obtained with a weight of 14.9 g and a purity of 8.1%. The sugar contents of WM-N and WM-A were determined to be 49% and 27%, respectively. The monosaccharide composition of WM-N and WM-A is shown in Table 4. As shown in Figure 3A, WM-N showed a wide distribution in *Mw*. Further purification will be carried out on WM-N and future research will focus on the purification and investigation of WM-A.

### 3.4. Fractionation by Dialysis

The 80 g mass of WM-N was subjected to additional purification using a dialysis bag with a *Mw* cut-off of 3500 Da and the resulting high *Mw* WM-NP’ weighed in at a relative 41.3 g. Compared with WM-N, the sugar content of WM-NP’ increased significantly, reaching a yield of 51.6%. Through the analysis of monosaccharide composition in Table 4, it was found that the contents of Glc and Ara in WM-NP’ further increased, while the contents of Gal and Glc decreased. By comparing the HPGPC chromatogram in Figure 3A,B, WM-NP’ showed a single symmetrical peak, which indicated that it was a homogeneous polysaccharide, and the presence of a sharp oligosaccharide peak in WM-N indicated that it was not a homogeneous polysaccharide before and after the grading. This study finds that the homogeneity of WM-NP’ *Mw* distribution was higher than that of WM-N but the *Mw* distribution was still broad and can be further purified according to its *Mw*.

### 3.5. Fractionation by Alcohol Precipitation

The WM-NP’ was purified by the ethanol precipitation method to improve its relative *Mw* uniformity and sugar content. Optimization of the alcohol precipitation conditions was optimized. After precipitating with ethanol at final concentrations of 40%, 50%, 60%, and 70%, four distinct samples were obtained: WM-NP’-40, WM-NP’-50, WM-NP’-60, and WM-NP’-70. The weight of each sample was weighed and the sugar content and relative *Mw* were determined. By comparison, it was found that the yield of WM-NP’-70 was the highest, while that of WM-NP’-40 was the lowest. This was because the higher the relative *Mw* of polysaccharides, the lower their solubility in alcohol. A higher concentration of ethanol could precipitate most of the polysaccharides with a wider *Mw* distribution. From the perspective of sugar content, WM-NP’-60 had the highest sugar content, followed by WM-NP’-50. The results are shown below in Table 5.

In terms of relative *Mw*, it could be observed from Figure 3B that WM-NP’-40, WM-NP’-50, WM-NP’-60, and WM-NP’-70 were all single symmetrical peaks, indicating that they were all homogeneous polysaccharides. Among them, WM-NP’-60 showed a good normal distribution; the uniformity of WM-NP’-60 was the best. Based on the yield, sugar content, and *Mw* uniformity, the ethanol concentration was 60%. According to the experimental data, the concentration of WM-NP’ polysaccharide solution was 10 mg/mL, 20 mg/mL, and 50 mg/mL, respectively. Finally, the sample with 60% ethanol concentration was obtained by the ethanol precipitation method. The weight of the precipitate was weighed and the yield was calculated to determine the sugar content in the polysaccharide precipitate. The results are shown below in Table 6.

It was discovered that as sample concentration was raised, polysaccharide production went up, while sugar content went down. Considering the analysis results, the WM-NP’ solution with a concentration of 10 mg/mL was finally determined as the purification condition. The specific purification method was to dissolve WM-NP’ in 60% ethanol and purify it as the final concentration. These experimental results provided important guidance for the purification process of WM-NP’.

Based on the HPLC analysis in Table 7, WM-NP’-60 were determined to be Glc (91%), Gal (3.8%), Man (4.2%), and Ara (1.0%). (Related Figures S4 and S5 are shown in the Supplementary Material). Furthermore, WM-NP’-60 had a relative *Mw* of 10 kDa. In general, *α*-glycosides are represented by positive rotation and *β*-glycosides by negative rotation [34]. At a temperature of 28.7 °C, the optical rotation of WM-NP’-60 was +0.049. These data provided important information about WM-NP’-60.

### 3.6. Periodate Oxidation and Smith Degradation

**Figure 4:**
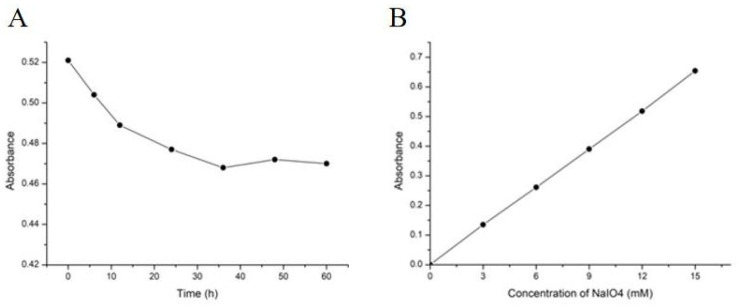
(**A**) Curve of sodium periodate consumption over time; (**B**) Standard curve of sodium periodate (*y* = 0.0433*x* + 0.0014, *R*^2^ = 0.9999).

The glycosidic bond type of WM-NP’-60 was analyzed. Glc was mainly present and a 50 mg sample contains 0.31 mM of sugar residues. After 60 h of periodate oxidation reaction, it reached equilibrium. The consumption of periodate and formic acid production was determined. The results showed that the absorption value of the sample at the completion of the reaction was 0.278 (Figure 4A). Based on the alternative standard curve (Figure 4B), the concentration of sodium periodate at the end of the reaction was calculated to be 6.4 mM. Therefore, the total consumption of sodium periodate was 0.43 mM. According to the titration experiment with sodium hydroxide, it was found that 0.22 M of formic acid was formed. This indicated that per mole of residue, 1.39 M of sodium periodate was consumed to produce 0.71 M of formic acid. The consumption of sodium formate and sodium periodate was approximately twice that of formic acid. This suggested that the glycosidic bonds destroyed by periodic acid were of the type 1→ or 1→6, instead of 1→2, 1→2, 6, 1→4, and 1→4, 6, and only key types of periodate were consumed without producing formic acid. Additionally, 70% of sugar residues were oxidized, indicating that there were some bond types that periodate could not oxidize, such as 30% of 1→3, 1→3, 6, 1→2, 3, 1→2, 4, and 1→3, 4.

**Figure 5:**
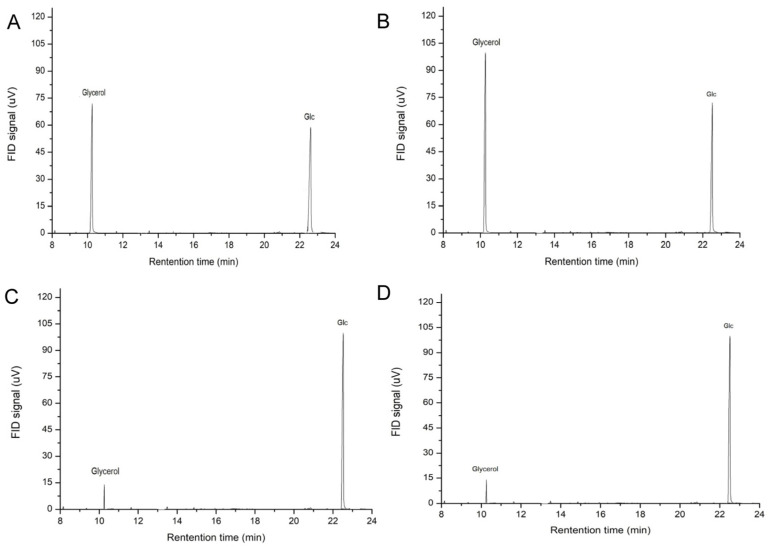
(**A**) GC spectrum of complete acid hydrolysis of the reduced product, (**B**) GC spectrum of the dialysis bag external fluid, (**C**) GC spectrum of liquid alcohol precipitation supernatant in a dialysis bag, (**D**) GC spectrum of liquid alcohol precipitation in a dialysis bag.

KBH_4_ further reduces the oxidation products of periodate and then hydrolyzes under the action of dilute acid. According to GC detection, glycerol and Glc were found in the hydrolysate, but Erythrose was not detected (refer to Table 8 and Figure 5). These results indicated that WM-NP’-60 contained 1→ and 1→6 linkages that could form glycerol, as well as other linkages such as 1→3 and 1→3, 6 that were not oxidized by iodate. After degradation treatment, Glc (glucose) could be detected in ethanol precipitation, supernatant, and precipitation in the bag. This suggested that there were Glucan fragments with different degrees of polymerization.

Based on the analysis results of periodic acid oxidation and Smith degradation, important speculations about the structure of WM-NP’-60 have been made. It was speculated that this structure was mainly composed of 1,6-Glc, accounting for approximately 70% of the total structure. Additionally, this structure also contained 30% of 1,3-Glc and 1,3,6-Glc.

### 3.7. NMR Spectroscopy Analysis

**Figure 6:**
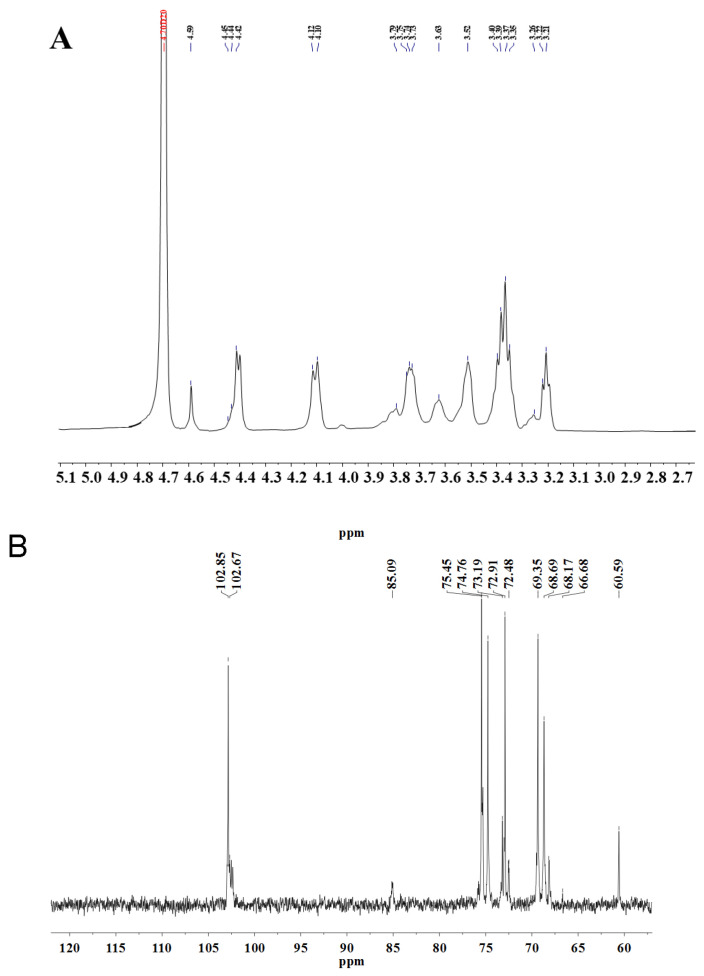
(**A**) ^1^H NMR spectrum of WM-NP’-60 and (**B**) ^13^C NMR spectrum of WM-NP’-60.

^1^H NMR spectra can provide hydrogen signals from sugar residue anomeric with hydrogen signals in other positions. It can be used to analyze individual sugar residue types. Typically, the δ 4.3–5.9 ppm is the region of anomeric hydrogen for polysaccharides, *α*-type sugar residues have anomeric hydrogens in excess of δ 5.0, and *β*-type sugar residues generally have anomeric hydrogens of less than δ 5.0 [35]. As shown in the ^1^H NMR spectrum of WM-NP’-60 (Figure 6A), the anomeric signal was observed to be attributed to *β*-type sugar residues. The signals of the anomeric carbons as well as the number of sugar residues can be observed in the ^13^C NMR spectra. In the ^13^C NMR of WM-NP’-60, there were six distinct signal peaks at δ 102.82 ppm, 69.38 ppm, 72.70 ppm, 69.38 ppm, 74.66 ppm, and 68.60 ppm, which were attributed to the C-1 to C-6 of *β*-1 and 6-D-Glc*p*, respectively. δ 85.02 ppm and 60.58 ppm were attributed to the C-3 and C-6 of *β*-1,3-D-Glc*p*, respectively. δ 68.01 ppm was the absorption signal peak of *β*-1,3,6-D-Glc*p* C-2 and the chemical shifts of the other carbons of *β*-1,3,6-D-Glc*p* may *β*-1,6-D-Glc*p* and *β*-1,3-D-Glc*p* overlap. The low content of Gal in WM-NP’-60 failed to find its associated peaks in NMR. With the attribution of other shift values based on the literature, the HSQC spectral chemical shift values are attributed in Table 9.

Figure 6 presented the ^1^H NMR and ^13^C NMR spectroscopy results for WM-NP’-60. These findings were consistent with the information documented in the previous literature [36,37,38,39,40,41,42,43]. In ^1^H NMR (Figure 6A), a peak of 4.59 ppm was observed corresponding to *β*-1,6-D-Glc*p* and *β*-1,3,6-D-Glc*p*. In ^13^C NMR, it was found that the peak of 102.67 ppm corresponds to *β*-1,6-D-Glc*p* and anomeric carbon signal of *β*-1,3,6-D-Glc*p*. In addition, the low field chemical shift of 74.76 ppm indicates that the C-3 position of Glc has been replaced. The peak of 85.09 ppm was attributed to *β*-1,3-D-Glc*p* or the C-3 position of *β*-1,3,6-D-Glc*p*. The peak of 60.59 ppm was attributed to *β*-1,3-D-Glc*p* or nonreducing terminal C-6. In addition, 75.45 ppm, 73.19 ppm, 69.35 ppm, and 68.69 ppm correspond to C-5, C-2, C-6, and C-4 of *β*-1,6-D-Glc*p.* In conclusion, it can be deduced that WM-NP’-60 primarily consists of *β*-1,6-D-Glc*p*, with a minor presence of *β*-1,3-D-Glc*p* or *β*-1,3,6-D-Glc*p*. The carbon resonance signal of WM-NP’-60 aligns with the monosaccharide composition.

**Figure 7:**
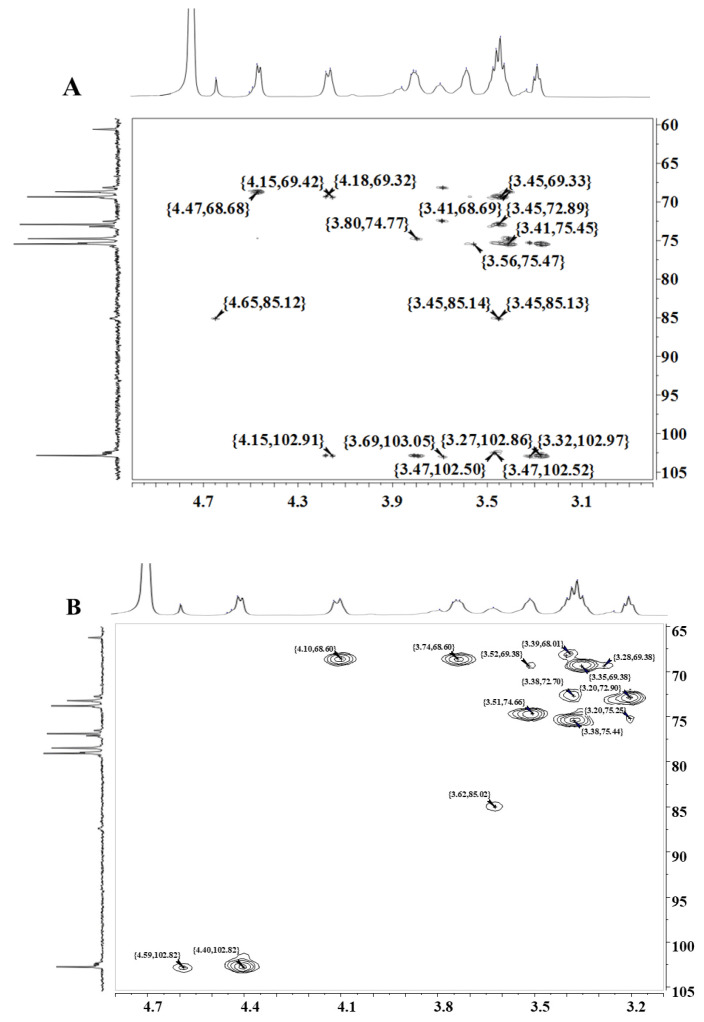
HMBC (**A**) and HSQC (**B**) spectrum of WM-NP’-60.

Combined with the HMBC results of WM-NP’-60 in Figure 7, the linkage connection order of sugar residues in WM-NP’-60 could be hypothesized. If *β*-1,6-D-Glc*p*, *β*-1,3-D-Glc*p*, and *β*-1,3,6-D-Glc*p* in WM-NP’-60 were named with the letters A, B, and C, the results showed the existence of the following coupling relationships: AC-1/AH6a/6b; BC-3/BH-1; CC-1/BH-3; or BC-1/BH-3. The presence of both linear *β*-1,6-glucan and *β*-1,3-glucan in WM-NP’-60 was confirmed and its structure might be based on the linear *β*-1,6-glucan as the backbone and a small amount of branching at the 3-position of the backbone sugar group attached to the linear *β*-1,3-glucan as the side chain. Additionally, a minor proportion of Gal may be linked either directly to the main chain as a side chain or to a side chain.

### 3.8. Methylation Analysis

Since WM-NP’-60 was a homogeneous neutral sugar grade obtained by systematic isolation and purification of sorghum Wumi polysaccharides, its structure was analyzed by methylation analysis. As can be seen from the Table 10, Glc residues in WM-NP’-60 were present as 2,3,4- Me3-Glc*p*, 2,4,6-Me3-Glc*p,* and 2,4-Me2-Glc*p*. The ratio of sugars was 77:21:2. No relevant peaks were detected due to the low Gal content.

**Figure 8:**
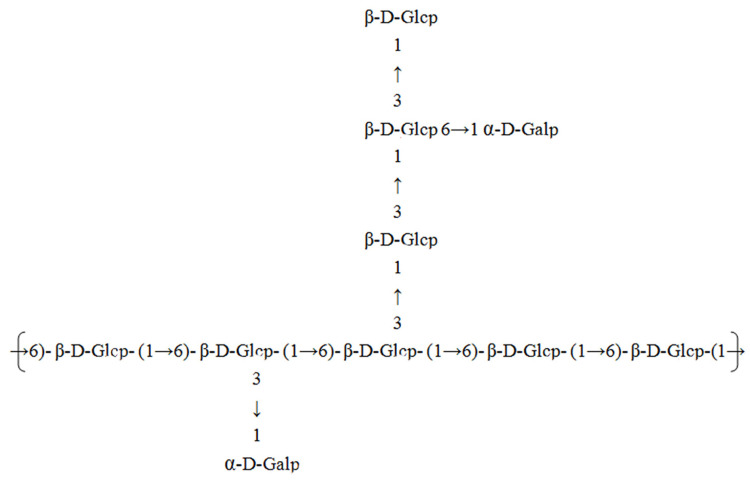
The predicated structure of WM-NP’-60.

According to the results of the monosaccharide composition determination, periodate oxidation, Smith degradation, NMR analysis, and methylation analysis, the structure of WM-NP’-60 was inferred in Figure 8. The main structure consists of *β*-1,6-D-Glc*p* forming the main chain, with side chains attached to the main chain via glycosyl bonds at the C-3 position. The side chain is made up of *β*-1,3-D-Glc*p*, while the glucan structure comprises *β*-1,3,6-D-Glc*p*.

### 3.9. Antioxidant Activity In Vitro

#### 3.9.1. Scavenging Activity to the DPPH Radical

**Figure 9:**
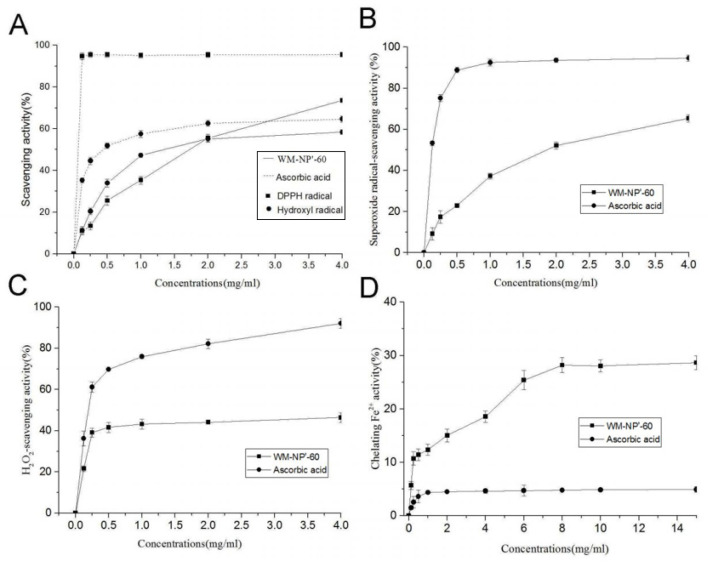
Antioxidant activity of WM-NP’-60 from the fruit bodies of *S. reilianum* (Fries): (**A**) scavenging activities to DPPH-radical and hydroxyl radical, (**B**) scavenging activities to superoxide anion, (**C**) scavenging activities to H_2_O_2_, and (**D**) chelating activity on ferrous ion.

The compounds’ hydrogen-donating properties are mostly responsible for their DPPH-scavenging efficacy. In Figure 9A, the scavenging activity of WM-NP’-60 varied as a function of concentration. The fact that its EC_50_ value for scavenging DPPH free radicals was so low (1.74 mg/mL) suggests that WM-NP’-60 is a potent antioxidant. However, its capacity to inhibit was diminished when compared to that of ascorbic acid.

#### 3.9.2. Scavenging Activity to the Hydroxyl Radical

In the presence of ascorbic acid, a pink glow was observed in the reaction solution when deoxyribose was subjected to heat and TBA in an acidic environment, resulting from the generation of hydroxyl radicals through the complexation of EDTA iron with H_2_O_2_. The amount of hydroxyl radicals can be estimated by looking at how pink the substance was. Figure 9A shows that WM-NP’-60′s ability to scavenged hydroxyl radicals varied with the concentration of the compound. Compared to ascorbic acid, WM-NP’-60 had a lower EC_50_ value, at 0.55 mg/mL. This showed that WM-NP’-60 was very effective at neutralizing hydroxyl radicals.

#### 3.9.3. Scavenging Activity to the Superoxide Anion

The ability to scavenge superoxide anions is crucial in the fight against oxidation. Figure 9B displays that WM-NP’-60 significantly reduced superoxide anion levels, with an EC_50_ of 0.95 mg/mL. While WM-NP’-60 showed lower scavenging activity compared to ascorbic acid, it displayed significant effectiveness in scavenging superoxide anions, suggesting its promising potential as an antioxidant agent.

#### 3.9.4. Scavenging Activity to H_2_O_2_

In the presence of free radicals, H_2_O_2_ acts as an intermediary for ROS molecules. According to Figure 9C, the scavenging capability of WM-NP’-60 against H_2_O_2_ rose as the concentration increased from 0.125 to 4 mg/mL, reaching a plateau at approximately 1 mg/mL. Experiments showed that WM-NP’-60 had some scavenging impact on H_2_O_2_, even though its scavenging activity was lower than that of ascorbic acid. These results suggest that WM-NP’-60 has the potential to be utilized as a tool to control the production of free radicals.

#### 3.9.5. Chelating Activity on the Ferrous Ion

The ferrous ions are highly reactive metal elements that can promote oxidative changes in cellular components. According to Figure 9D, the chelating ability of WM-NP’-60 was positively correlated with its concentration. Compared with ascorbic acid, its chelating ability was more prominent. At a dose of 8 mg/mL, the chelating activity reached 28.21% during the plateau period. The results showed that WM-NP’-60 has a certain chelating effect on ferrous ions.

### 3.10. ROS Detection in Cells

**Figure 10:**
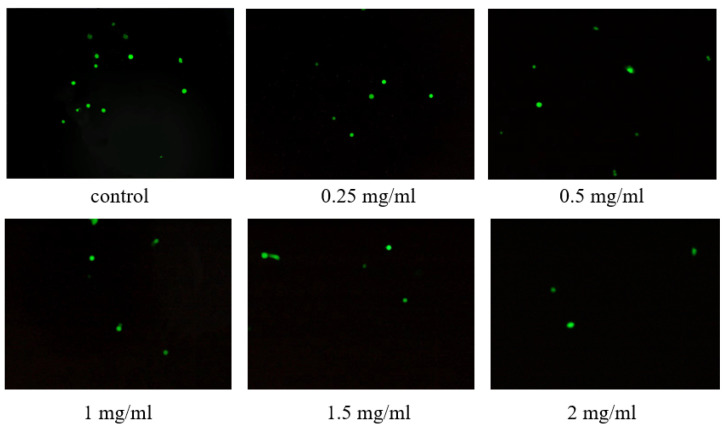
ROS levels in the NCM460 cells of each group (200×).

The DCFH-DA without fluorescence can freely cross the cell membrane and enter living cells and is then hydrolyzed to produce DCFH, which cannot easily pass through the cell membrane. In the presence of ROS, DCFH is oxidized to produce the fluorescent 2′,7′-dichlorofluorescein (DCF) [30]. According to Figure 10, in contrast to the control group, the ROS levels in cells gradually decreased with the increasing concentration from 0.25 to 2 mg/mL of WM-NP’-60.

There have been numerous studies on the biological properties of polysaccharides containing *β*-galactofuranose and *β*-glucopyranose residues, yet limited research exists on their antioxidant capabilities [44,45]. Certain polysaccharides rich in glucose or galactose are crucial in protecting organisms from oxidative harm by acting as scavengers for free radicals, chelating metal ions, and serving as reducing agents. Chelating activity means that the polysaccharides may can also bind other important ions in the cellular environment and non-specifically reduce cell proliferation [46,47]. As an illustration, polysaccharides derived from Ganoderma lucidum, featuring (1→2)-, (1→3)-, (1→4)-, and (1→6)-linked *β*-Glc*p* residues, demonstrate capabilities in scavenging free radicals, sialylation, and chelating ions [48]. WM-NP’-60 contains galactose and glucose residues and the results of the in vitro and in vivo antioxidant experiments in this study showed that WM-NP’-60 had significant antioxidant activity. Therefore, WM-NP’-60 can be considered as a potential natural antioxidant.

### 3.11. Antitumor Activity

#### 3.11.1. Anti-Proliferation Activity Assay

**Figure 11:**
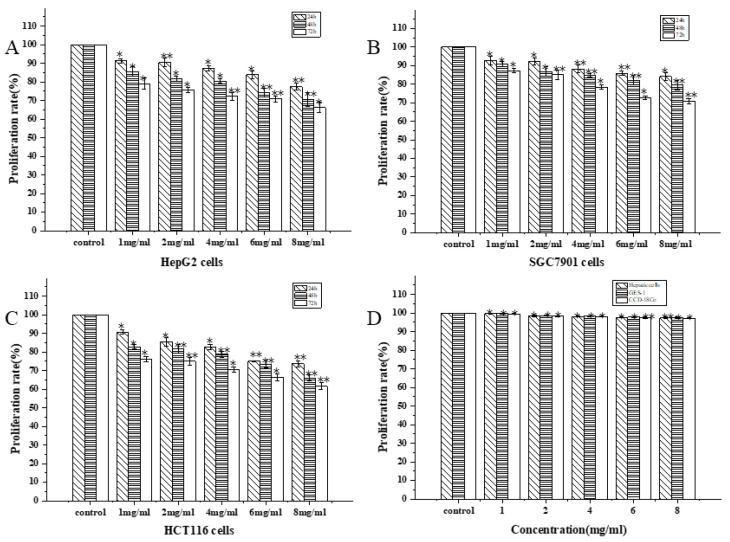
Effects of WM-NP’-60 on anti-proliferation of HepG2 cells (**A**), SGC7901 cells (**B**), HCT116 (**C**), and three normal cells (**D**). After 24, 48, and 72 h of cultivation in HepG2, SGC7901, and HCT116 cells and after 24 h of cultivation in three normal cells, the MTT method was used to evaluate the growth of WM-NP’-60 at different concentrations (1, 2, 4, 6, and 8 mg/mL). * *p* < 0.05 and ** *p* < 0.01.

Using the MTT assay allowed for a more precise evaluation of WM-NP’-60′s antitumor potential on HepG2, SGC7901, and HCT116 cells. HepG2, SGC7901, and HCT116 cells and three normal cells, including hepatic cells, GES-1, and CCD-18Co, were placed in different concentrations (1, 2, 4, 6, and 8 mg/mL) of WM-NP’-60. HepG2, SGC7901, and HCT116 cells were incubated for 24, 48, and 72 h and three normal cells were incubated for 24 h, respectively. The results showed that WM-NP’-60 inhibited the proliferation of HepG2, SGC7901, and HCT116 cell lines in a dose- and time-dependent manner (Figure 11). For HCT116 cells, WM-NP’-60 showed the most significant inhibitory effect, followed by its inhibitory effect on HepG2 cells and SGC7901 cells. WM-NP’-60 exhibited the highest sensitivity toward HCT116 cells. When the concentration was 8 mg/mL and the treatment time was 24 and 72 h, the survival rates of HepG2, SGC7901, and HCT116 cells were 77.46 ± 1.74%, 66.30 ± 2.77%, 84.12 ± 2.25%, 70.74 ± 1.45%, 73.63 ± 1.74%, and 61.58 ± 1.74% and the inhibition rates were 22.54 ± 1.74%, 33.70 ± 2.77%, 15.88 ± 2.25%, 29.26 ± 1.45%, 26.37 ± 1.74%, and 39.42 ± 1.74%, respectively. These results indicated that WM-NP’-60 has significant antitumor activity on HepG2, SGC7901 cells, and HCT116 cells. Figure 11D shows that the survival rates of the three normal cell types remained above 97% at different concentrations.

The antitumor activity of fungal polysaccharides was significantly influenced by the monosaccharide composition [49]. In general, certain types of fungal polysaccharides that were abundant in glucose, galactose, or mannose were found to possess significant antitumor properties [50,51]. WM-NP’-60 has structural features of fungal polysaccharides and it was sensitive to HepG2, SGC7901, and HCT116 cells, which can be regarded as a natural compound with potential anticancer properties.

#### 3.11.2. TEM Assay

**Figure 12:**
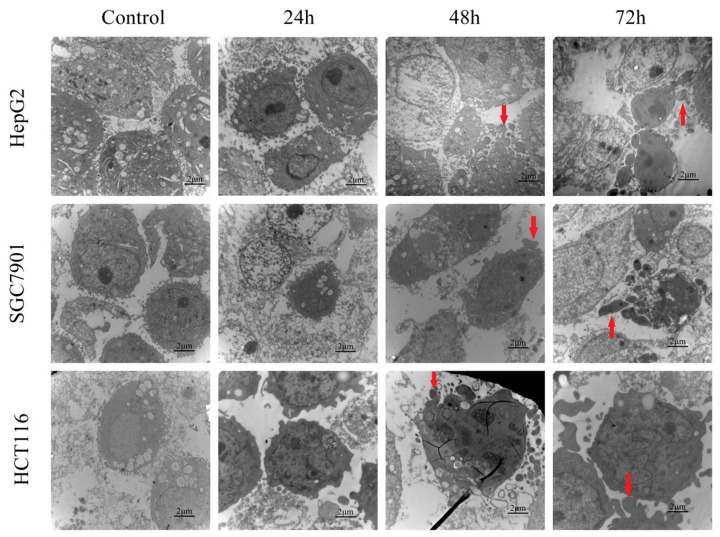
TEM of HepG2 cells, SGC7901 cells, and HCT116 cells treated with WM-NP’-60 at 4 mg/mL. The red arrow points to the appearance of apoptotic bodies.

Apoptotic morphology induction by WM-NP’-60 (4 mg/mL) on HepG2 cells, SGC7901 cells, and HCT116 cells was observed by transmission electron microscopy (TEM) examination (Figure 12). Significant differences were seen between the treated and control cells after 24, 48, and 72 h of incubation. The edge of the cytoplasm became rough, membrane bubbles formed, and cell contraction and chromatin concentration appeared. At the end, a typical apoptotic body structure was formed.

## 4. Conclusions

In conclusion, the polysaccharide (WM-NP’-60) extracted from diseased sorghum heads by *Sporisorium reilianum* possess a high value and the investigation of their structure and biological activities is crucial for future applications. Monosaccharide composition, periodate oxidation, Smith degradation, NMR, and methylation analysis revealed that a C-3 glycosyl bond joined the main chain of *β*-1,6-D-Glc*p* to the side chain of the main chain in WM-NP’-60. The glucan side chain structures of *β*-1,3-D-Glc*p* and *β*-1,3,6-D-Glc*p* were present. Research on the antioxidant properties of WM-NP’-60 demonstrates its great antioxidant activity both in cell and in vitro. Anti-proliferation assays and observations of characteristic apoptotic bodies in response to HepG2 cells, SGC7901 cells, and HCT116 cells provide experimental proof that WM-NP’-60 demonstrates considerable anti-tumor effects in vitro. It shows substantial promise as antioxidants and anti-tumor agents based on current scientific evidence. However, there are limitations in this study. The bioactivity of WM-NP’-60 at relatively high concentrations was investigated in this study. The experiments demonstrated that WM-NP’-60 exhibited substantial bioactivities, including antioxidant and antitumor effects at relatively high concentrations. However, the form in which this polysaccharide is introduced into the body will be the subject of further investigation in the future. Additionally, the acidic sugar WM-A represents a direction for future research. Further elucidation of the molecular mechanisms underlying the antioxidant and anti-tumor properties of WM-NP’-60 is essential and deeper exploration into hypoglycemic and lipid-lowering effects in future research is necessary. This will enable it to become valuable targets for future endeavors aimed at safeguarding human health.

## Figures and Tables

**Table 1:** Factors and levels for orthogonal test design.

Levels	Factors			
	A (Temperature, °C)	B (Time, min)	C (Solid–Liquid Ratio)	D (Frequency)
1	70	120	1:10	2
2	80	180	1:20	3
3	90	240	1:40	4

**Table 2:** Results from orthogonal test design.

No.	Factors				Yield, %
	A (Temperature, °C)	B (Time, min)	C (Solid–Liquid Ratio)	D (Frequency)	
1	70	120	1:10	2	5.06
2	70	180	1:20	3	5.50
3	70	240	1:40	4	5.83
4	80	120	1:20	4	6.37
5	80	180	1:40	2	6.50
6	80	240	1:10	3	6.30
7	90	120	1:40	3	7.22
8	90	180	1:10	4	7.12
9	90	240	1:20	2	7.14
k_1_	5.46	6.22	6.16	6.23	-
k_2_	6.39	6.37	6.34	6.34	-
k_3_	7.16	6.42	6.52	6.44	-
R	1.70	0.20	0.36	0.21	-
OK	A_3_	B_3_	C_3_	D_3_	-

**Table 3:** Monosaccharide composition of WM.

Fraction	Monosaccharide Composition (%)					
	Glc	Man	Gal	Ara	Rha	GlaA
WM	69.6	15	11	1.9	1.7	0.8

**Table 4:** Monosaccharide composition of WM-A, WM-N, and WM-NP’.

Fraction	Monosaccharide Composition (%)				
	Glc	Gal	Man	Ara	GalA
WM-A	66.89	16.29	6.83	4.56	5.43
WM-N	80.05	10.96	8.22	0.77	-
WM-NP’	87.50	9.50	1.40	1.60	-

**Table 5:** Determination of alcohol precipitation concentration of WM-NP’.

Sample Concentration (mg/mL)	Alcohol Concentration (%)	Yield (%)	Sugar Content (%)
10	40	28.0	66.0
10	50	44.0	84.0
10	60	67.4	86.8
10	70	83.0	85.0

**Table 6:** Determination of the sample concentration of WM-NP’.

Sample Concentration (mg/mL)	Alcohol Concentration (%)	Yield (%)	Sugar Content (%)
10	60	67.4	87.0
20	60	73.5	75.2
50	60	84.5	69.3

**Table 7:** Comparison of WM-NP’-60 monosaccharide composition.

Fraction	Monosaccharide Composition (%)			
	Glc	Gal	Man	Ara
WM-NP’-60	91	3.8	4.2	1.0

**Table 8:** GC results of Smith-degradation fractions of WM-NP’-60.

	Full Acid Hydrolysis	Out of Sack	Supernatant in Sack	Precipitation in Sack
Glc	+ ^a^	+	+	+
Gly	+	+	+	+
Ery	− ^b^	−	−	−

Note: Glc = Glucose; Gly = Glycerol; Ery = Erythritol. ^a^ Detectable. ^b^ Undetectable.

**Table 9:** Chemical shift attribution of the HSQC spectrum of WM-NP’-60.

Type		1	2	3	4	5	6
*β*-1,6-D-Glc*p*	H	4.59	3.28	3.38	3.52	3.51	4.10, 3.74
	C	102.82	69.38	72.70	69.38	74.66	68.60
*β*-1,3-D-Glc*p*	H	4.41	3.35	3.62	3.52	3.51	3.80; 3.62
	C	102.82	69.38	85.02	69.38	74.66	60.58
*β*-1,3,6-D-Glc*p*	H	4.59	3.39	3.62	3.52	3.51	4.10; 3.74
	C	102.82	68.01	85.02	69.38	74.66	68.60

**Table 10:** Methylation results of WM-NP’-60.

Methylated Sugar	Connection Type	Percentage (%)	Fragmentation Ion Peak
2,3,4-Me3-Glc*p*	1,6-Glc	77	87, 101.1, 117, 129, 161.1, 173, 189.1, 233.1
2,4,6-Me3-Glc*p*	1,3-Glc	21	87, 101.1, 117, 129, 143, 161.1, 173, 233.1
2,4-Me2-Glc*p*	1,3,6-Glc	2	87, 97, 117, 129, 189, 233.1, 305.1

## Data Availability

The dataset presented in this article is available upon request.

## Supplementary Materials

The following supporting information can be downloaded at: https://www.mdpi.com/article/10.3390/antiox13080965/s1. Figure S1: Total sugar standard curve. Figure S2: Protein standard curve. Figure S3: Galacturonic acid standard curve. Figure S4: Monosaccharide composition of WM-NP’-60 by HPLC. Figure S5: The full wavelength scanning of WM-NP’-60. Table S1: Sugar content analysis of crude WM. Table S2: Protein content analysis of crude WM. Table S3: Galacturonic acid content analysis of crude WM.
