# Supplementary material for: Extraction Optimization, Structural Analysis, and Potential Bioactivities of a Novel Polysaccharide from Sporisorium reilianum
Source: Antioxidants (Basel). 2024 Aug 8;13(8):965. doi: 10.3390/antiox13080965 (PMC11352142; doi:10.3390/antiox13080965)
Supplement: Supplementary file 1 [file antioxidants-13-00965-s001.zip › antioxidants-3127717-supplementary.pdf]

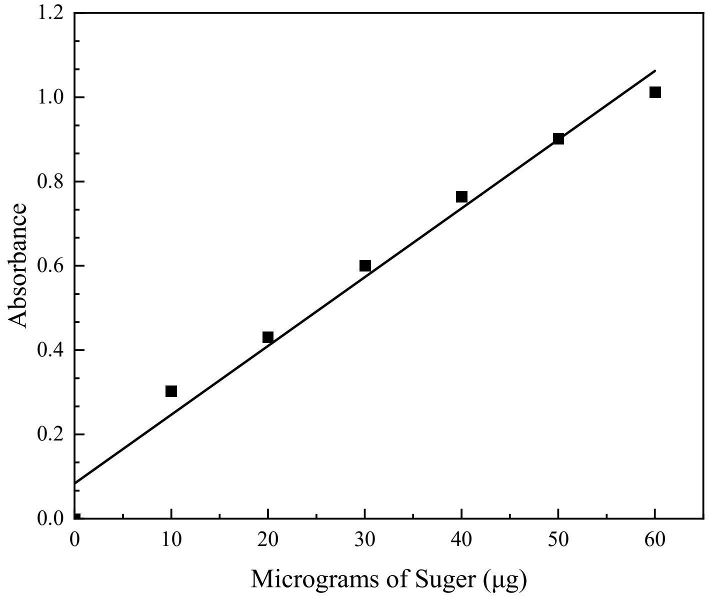


**Figure S1**. Total sugar standard curve.

**Table S1.** Sugar content analysis of crude WM.

| Sample | | Absorbance Value | Mean Absorbance Value | Sugar Content |
|---|---|---|---|---|
| WM | 1 | 0.472 | | |
| WM | 2 | 0.442 | 0.471±2.90% | 47.8%±2.90% |
| WM | 3 | 0.500 | | |

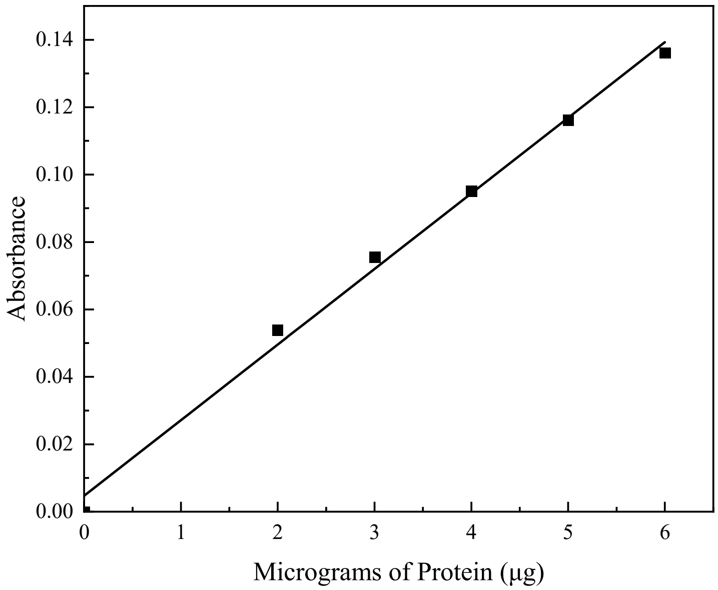


**Figure S2.** Protein standard curve.

**Table S2**. Protein content analysis of crude WM.

| Sample | | Absorbance Value | Mean Absorbance Value | Sugar Content |
|---|---|---|---|---|
| WM | 1 | 0.062 | | |
| WM | 2 | 0.058 | 0.06±0.58% | 1.2%±0.58% |
| WM | 3 | 0.060 | | |

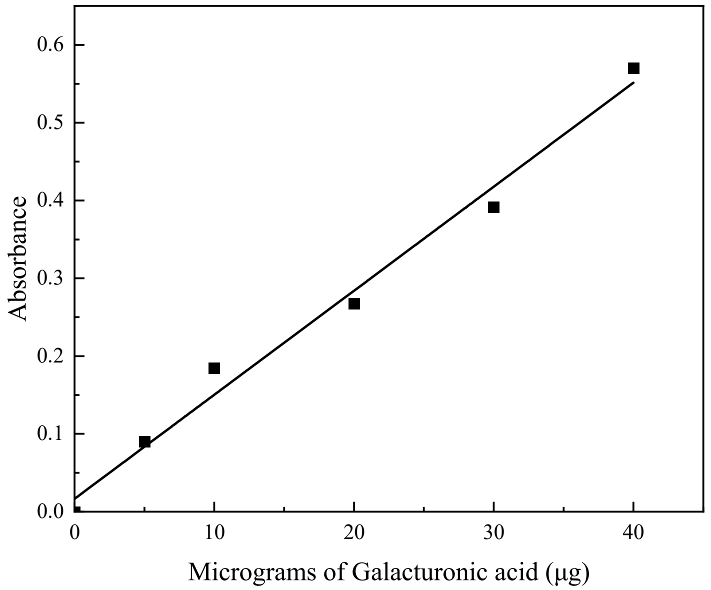


**Figure S3.** Galacturonic acid standard curve.

**Table S3**. Galacturonic acid content analysis of crude WM.

| **Sample** | | **Absorbance Value** | **Mean Absorbance Value** | **Sugar Content** |
|---|---|---|---|---|
| WM | 1 | 0.055 | | |
| WM | 2 | 0.057 | 0.056±0.10% | 6.09%±0.10% |
| WM | 3 | 0.056 | | |

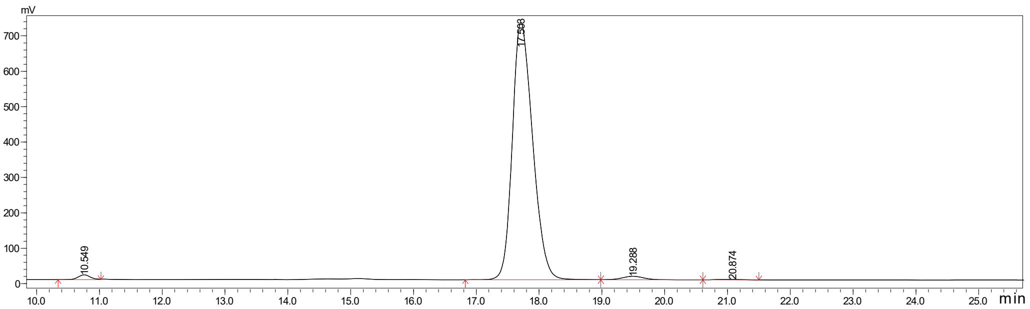


**Figure S4.** Monosaccharide composition of WM-NP'-60 by HPLC.

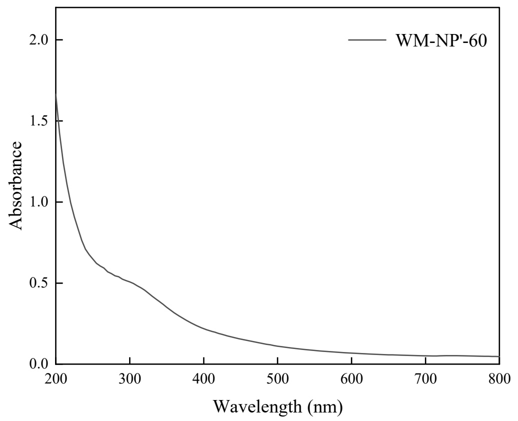


**Figure S5**. The full wavelength scanning of WM-NP'-60.
